# Correct and stable sorting for overflow streaming data with a limited storage size and a uniprocessor

**DOI:** 10.7717/peerj-cs.355

**Published:** 2021-02-12

**Authors:** Suluk Chaikhan, Suphakant Phimoltares, Chidchanok Lursinsap

**Affiliations:** Advanced Virtual and Intelligent Computing (AVIC) Research Center, Department of Mathematics and Computer Science, Faculty of Science, Chulalongkorn University, Bangkok, Thailand

**Keywords:** Algorithms, Sorting, Memory, Algorithm design and analysis, Computational intelligence

## Abstract

Tremendous quantities of numeric data have been generated as streams in various cyber ecosystems. Sorting is one of the most fundamental operations to gain knowledge from data. However, due to size restrictions of data storage which includes storage inside and outside CPU with respect to the massive streaming data sources, data can obviously overflow the storage. Consequently, all classic sorting algorithms of the past are incapable of obtaining a correct sorted sequence because data to be sorted cannot be totally stored in the data storage. This paper proposes a new sorting algorithm called *streaming data sort* for streaming data on a uniprocessor constrained by a limited storage size and the correctness of the sorted order. Data continuously flow into the storage as consecutive chunks with chunk sizes less than the storage size. A theoretical analysis of the space bound and the time complexity is provided. The sorting time complexity is *O* (*n*), where *n* is the number of incoming data. The space complexity is *O* (*M*), where *M* is the storage size. The experimental results show that *streaming data sort* can handle a million permuted data by using a storage whose size is set as low as 35% of the data size. This proposed concept can be practically applied to various applications in different fields where the data always overflow the working storage and sorting process is needed.

## Introductions

Currently, the growth of data consumption by internet users has exponentially increased (Laga et al., 2017; Bey Ahmed Khernache, Laga & Boukhobza, 2018), and a massive storage size is required to store all incoming data to avoid any data loss in case of storage overflow (Thusoo et al., 2010; Katal, Wazid & Goudar, 2013; Witayangkurn, Horanont & Shibasaki, 2012; Mehmood et al., 2016). However, many applications such as data management, finance, sensor networks, security-relevant data, and web search possibly face this unexpected situation of a storage overload issue (Lee et al., 2016; Babcock et al., 2002; Keim, Qu & Ma, 2013; Cardenas, Manadhata & Rajan, 2013; Dave & Gianey, 2016). This issue induces the problem of representing big data with a limited storage size. Furthermore, some primitive operations such as the classic sorting algorithms (e.g., quick sort, heap sort) cannot be implemented due to the restrictive constraint of storing all sorted data inside the storage during the sorting process. A sorting algorithm is the first important step of many algorithms (Cormen et al., 2009; Huang, Liu & Li, 2019) such as searching and finding a closest pair (Singh & Sarmah, 2015; Tambouratzis, 1999).

Generally, when referring to data storage of a computer, it can be either primary storage (internal storage) or secondary storage (external storage). The size of primary storage is much smaller than that of the secondary storage. With reference to the size of storage, there are two types of sorting: internal sort and external sort. All data to be sorted by an internal sorting algorithm must be entirely stored inside the primary storage. Some of traditional internal sorting algorithms are bubble sort, insertion sort, quick sort, merge sort, and radix sort. However, if the data overflow the primary storage, the overflow must be stored in the secondary storage. In this case, external sort algorithms can be employed. Although these classic sorting algorithms are very efficient in terms of time and space complexities, the actual quantity of data generated yearly on the internet has grown tremendously faster than the growth rate of storage capacity based on the current fabrication technology (for both primary storage and secondary storage). This severe condition makes the classic sorting algorithms, where all data must be stored inside the computer, very inefficient because all overflowed data are lost.

In this study, both internal and external storage are viewed as one unit of storage with a limited size. This size is not gradually augmented during the sorting process of continuously incoming data. The challenging problem to be studied is how to sort the data under the constraints of limited storage capacity and storage overflow. The data are assumed to flow into the storage as a sequence of data chunks with various sizes less than or equal to the storage size.

Recently, many internal sorting algorithms have been remodeled by reducing comparison, swapping, and the time complexity to reduce the sorting time. Farnoud, Yaakobi & Bruck (2016) proposed an algorithm that sorts big data based on limited internal storage, but the result is a partial sort. Concoms sort (Agrawal & Sriram, 2015) is an algorithm that uses a swapping technique with no adjacent swapping. It reduces the execution time in some cases when compared to selection sort and outperforms bubble sort in every case. In particular, in the case that the input is a descending sequence, Concoms sort is more efficient than both traditional algorithms. Mapping sort (Osama, Omar & Badr, 2016) is a new algorithm that does not use comparisons and the swapping technique but it uses the mapping technique instead. This algorithm achieved the worst case time complexity of *O*(*n*) + *O*(*n* log *n*). Vignesh & Pradhan (2016) proposed a sorting algorithm by improving merge sort. It uses multiple pivots to sort data. The execution time of this algorithm is better than quick sort and merge sort in the best case and the average case, respectively. In addition, proximity merge sort (Franceschini, 2004) was proposed by improving the algorithm with an in-place property. Faro, Marino & Scafiti (2020) modified insertion sort to reduce the time complacency by inserting multiple elements for one iteration. The time complexity is $O({n^{1 + {1 \over h}}})$, where $h \in {{\mathbb N}}$. Idrizi, Rustemi & Dalipi (2017) modified the sorting algorithm by separating the data sequence into three parts, namely, negative numbers, zero numbers, and positive numbers. After the data in each part are sorted by printing the result, the algorithm can decrease the comparison by a separating process. Bidirectional conditional insertion sort algorithm (Mohammed, Amrahov & Çelebi, 2017) is a two-pivot insertion sort algorithm using the left comparator and right comparator. It is faster than insertion sort, and the time complexity is nearly close to *O*(*n*^1.5^). Brownian motus and clustered binary insertion sort methods (Goel & Kumar, 2018) are algorithms that adapted insertion sort and binary insertion sort to reduce the comparison and the execution time. Both algorithms are suitable for sorting partial data. Internal sorting algorithms in the literature have focused on reducing the time for processing, but the storage issue for big data has been ignored.

Presently, accessing a large piece of information or big data is simple because of rapid technological advancements such as the cloud (Al-Fuqaha et al., 2015; Kehoe et al., 2015; Vatrapu et al., 2016) and network technology (YiLiang & Zhenghong, 2016; Zhao, Chang & Liu, 2017; Zhai, Zhang & Hu, 2018). One of the issues for sorting big data is the restricted internal storage, which is usually smaller than the size of big data. All big data cannot be stored in the internal storage. Therefore, the internal sorting algorithms cannot sort big data at one time. The external sorting algorithms are developed from the classic merge sorting algorithm to sort big data, which is separated into two phases: (1) the sorting phase sorts a small chunk of big data in the internal storage. After sorting, all sorted chunks are stored in the external storage and (2) the merging phase combines all sorted chunks from the sorting phase into a single sorted list.

Recently, TaraByte sort (O’Malley, 2008) has used three Hadoop applications, namely, TeraGen, TeraSort, and TeraValidate, to sort big data. This algorithm sorts 10 billion data in 209 s. This process is very fast, but it is expensive because it requires many processing units for sorting. Kanza & Yaari (2016) studied external sorting problems and designed multi-insertion sort and SCS-Merge V1 to V3. The objective of these algorithms is to decrease the write cost of intermediate results of sorting. Active sort (Gantz & Reinsel, 2012) is an algorithm that merges sorted chunks inside SSDs and is applied with Hadoop to reduce the number of reading and writing data. MONTRES (Laga et al., 2017), the algorithm designed for SSDs, can reduce the read and write cost of I/O in a linear function. External sorting algorithms in the literature have focused on reducing the read and write cost in terms of the execution time for processing, but the storage issue for keeping big data is still ignored. Liang et al. (2020) proposed a new algorithm, namely, B*-sort, which was designed on NVRAM and applied on a binary search tree structure.

In addition, Farnoud, Yaakobi & Bruck (2016) studied approximate sorting of streaming permuted data with limited storage; however, the result is not exactly sorted data, and only an approximate result is obtained when determining the data positions. Conversely, the approximate positions of ordered data can be provided when using the values as inputs. Elder & Goh (2018) studied permutation sorting by finite and infinite stacks. Although all possible permutations cannot be sorted, the exact order and values can be obtained. Let *n* be the total streaming numbers to be sorted and *M* ≪ *n* be the limited size of working storage. Table 1 summarizes the efficiency of various classic sorting methods and our proposed method (stream sort) in terms of seven characteristics: requiring extra storage, preserving the input appearance order, time complexity, space complexity, sorting streaming data, correct sorting order, and correct retrieved value by the sorted order.

**Table 1:** Comparison of sorting algorithms on streaming data. *n* is the total streaming numbers to be sorted and *M* ≪ *n* is the limited size of working storage.

Sorting algorithms	Requiring extra storage	Preserving input appearance order	Time complexity	Working space complexity	Applicable to streaming data	Correct order	Correct value
Bubble sort	No	Yes	*O*(*n*^2^)	*O*(*n*)	No	Yes	Yes
Selection sort	No	No	*O*(*n*^2^)	*O*(*n*)	No	Yes	Yes
Insertion sort	No	Yes	*O*(*n*^2^)	*O*(*n*)	Yes	Yes	Yes
Quick sort	No	No	*O*(*n*^2^)	*O*(*n*)	No	Yes	Yes
Merge sort	Yes	Yes	*O*(*n* lg *n*)	*O*(*n*)	No	Yes	Yes
Heap sort	No	No	*O*(*n* lg *n*)	*O*(*n*)	No	Yes	Yes
Permutation sort (Farnoud, Yaakobi & Bruck, 2016)	No	No	*O*(*n*/*ω*(log^2^ *n*))	*O*(*n*)	No	Yes	Yes
Permutation sort (Elder & Goh, 2018)	Yes	Yes	N/A	*O*(*n*)	Yes	No	No
External sorting	Yes	Yes	N/A	*O*(*n*)	Yes	Yes	Yes
Streaming data sort	No	Yes	*O*(*n*)	*O*(*M*)	Yes	Yes	Yes

This article proposes a new algorithm called *streaming data sort* for sorting streaming data with limited storage size by using only a single central processing unit. The proposed algorithm can correctly and stably handle a streaming data size of at least 2.857 times larger than the size of the working storage. The following concerns are emphasized in this study.

- All data must be in the exactly correct order after being sorted. No approximate and partial ordering is allowed in this study.
- The time complexity of *streaming data sort* of all iterations is *O*(*n*).

## Constraints

**Figure 1:**
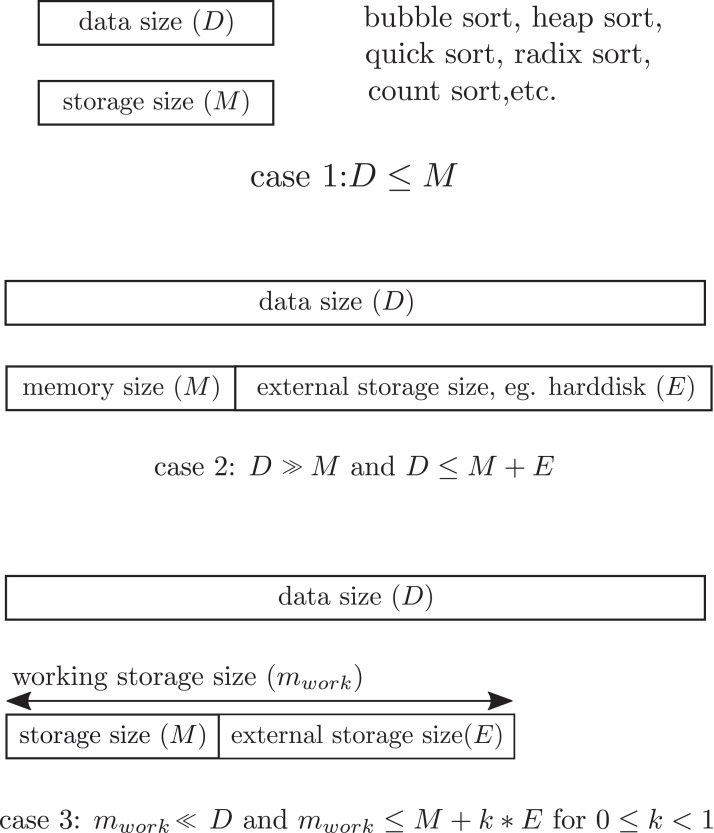
Storage constraint. Case 1 for *D* ≤ *M* where all data must be in the storage. Case 2 for *D* ≫ *M* and *D* ≤ *M* + *E* where data overflow the storage. Case 3 for *m*_work_ = *M* + *E*, the constraint of this study.

In the stationary data environment, all classic sorting algorithms are based on the assumption that all numbers to be sorted must be entirely stored in the working storage of a computer during the sorting process. This implies that the whole data set cannot exceed the working storage size during the sorting process. Figure 1 illustrates storage constraint of the working storage in *streaming data sort*. However, in the streaming data environment, the data continuously flow into the computer one chunk at a time, and the number of incoming chunks is unknown in advance. If the size of the data chunk is larger than the working storage size, then the overflow will be permanently discarded from the computer. This makes the sorted result wrong. To make the study sufficiently feasible for analysis and practice, the following constraints are imposed.

1. The sorting process is performed by using only a fixed working storage of size *M*. This working storage is for storing the incoming data, previously sorted data, and other temporal data structures generated during the sorting process. No extra storage module is added during this period. The proposed sorting algorithm and the operating system are not stored in this working storage.
2. All numbers are integers. For floating numbers, they must first be transformed into integers.
3. At any time *t*, the sizes of previously sorted data in a compact form and the size of next incoming data chunk (*h*) must not exceed *M*.
4. The present incoming data chunk is completely discarded after being processed by the proposed sorting algorithm.
5. Only four types of relation between any two temporal consecutive numbers *d*_*i*_ and *d*_*i*__+ 1_ are studied in this paper. The details and rationale of concentrating on these four types will be elaborated later.

The second constraint is the main concern of this study. After sorting the first incoming data chunk, all numbers are captured in a compact form and all sorted numbers are completely discarded. This compact form is used in conjunction with the next incoming data chunk for sorting. To avoid a storage overflow obstruction, the fourth constraint must be presented. The last constraint is derived from real-world data sets. From the observation of real-world streaming data sets from the UCI Repository (Dua & Graff, 2019) such as the census income, diabetes 130-US hospitals, incident management process event log, PM2.5 of five Chinese cities, KEGG metabolic relation network, Beijing multi-site air quality, and Buzz in social media, it is remarkable that most of the different values between two temporal consecutive numbers are between 0.38 and 2.98 on average. Hence, only four types of relations between any two temporal consecutive numbers are the focus. The definition of each type will be given in the next section.

## Definitions and Notations

**Definition 1** *The window at time t, denoted by* 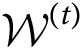 *= (d*_1_, *d*_2_, *…, d*_*h*_*), is a sequence of h ≤ M incoming numeric data at time t*.

**Definition 2** *The sorted window of* 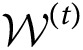 *at time t, denoted by W*^(*t*)^ *= (w*_1_, *w*_2_, *…, w*_*h*_ *| w*_*i*_ *= d*_*j*_, *w*_*i +* 1_ = ${d_k}\,\,{\kern 1pt} and{\kern 1pt} \,\,\forall {w_i},{w_{i + 1}} \in {W^{(t)}}:\,{w_i} < {w_{i + 1}})$, *is a sequence of increasingly sorted numeric data of* 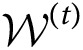.

**Definition 3 Type-1** *subsequence T*_*1*_ *= (w*_*i*_, *…, w*_*i + l*_*)* $\subseteq {W^{(t)}}$ *is a sequence such that ∀ w*_*i*_, *w*_*i +* 1_ *∈ T*_1_*: |w*_*i*_ *− w*_*i +* 1_*|* = 1.

An example of a Type-1 sequence is (1, 2, 3, 4, 5). The different value between any two adjacent numbers is equal to 1, namely, (|1 − 2|,|2 − 3|,|3 − 4|,|4 − 5|) = (1,1,1,1).

**Definition 4 Type-2** *subsequence T*_2_ = (*w_i_*, …,*w_i_*_+*l*_) ⊆*W*^(*t*)^ *is a sequence such that* ∀*w_i_*_+*a*_,*w_i_*_+*a*+1_ ∈ *T*_2_, 0 ≤ *a* ≤ *l*−1 : |*w_i_*_+*a*_−*w_i_*_+*a*+1_| = 1 *when a is even and* |*w_i_*_+*a*_−*w_i_*_+*a*+1_| = 2 *when a is odd*.

An example of a Type-2 sequence is (4, 5, 7, 8, 10). The different value between any two adjacent numbers is equal to either 1 or 2, namely, (|4–5|, |5–7|, |7–8|, |8–10|) = (1, 2, 1, 2).

**Definition 5 Type-3** *subsequence T*_3_ = (*w_i_*, …,*w_i_*_+*l*_) ⊆*W*^(*t*)^ *is a sequence such that* ∀*w_i_*_+*a*_,*w_i_*_+*a*+1_ ∈ *T*_3_, 0 ≤ *a* ≤ *l*−1 : |*w_i_*_+*a*_−*w_i_*_+*a*+1_| = 2 *when a is even and* |*w_i_*_+*a*_−*w_i_*_+*a*+1_| = 1 *when a is odd*.

An example of a Type-3 sequence is (5, 7, 8, 10, 11). The different value between any two adjacent numbers is equal to either 1 or 2, namely, (|5–7|, |7–8|, |8–10|, |10–11|) = (2, 1, 2, 1).

**Definition 6 Type-4** *subsequence T*_4_ = (*w_i_*,…,*w_i_*_+*l*_) ⊆ *W*^(*t*)^ *is a sequence such that* ∀*w_i_*,*w_i_*_+1_ ∈ *T*_4_: |*w_i_*−*w_i_*_+1_| = 2.

An example of a Type-4 sequence is (8, 10, 12, 14, 16). The different value between any two adjacent numbers is equal to either 1 or 2, namely, (|8–10|, |10–12|, |12–14|, |14–16|) = (2, 2, 2, 2).

During the sorting process by the proposed algorithm, it is necessary to identify the type of subsequence to be sorted first. Given a subsequence (*w_i_*,…,*w_i_*_+*l*_) ∈*W*^(*t*)^, the type of this subsequence can be easily identified as type-*p* by setting

(1) $$p = w_{i+2}+w_{i+1}-2(w_i+1)$$

Each already sorted subsequence (*w*_*i*_, …, *w*_*i*__+__*l*_) ∈ *W*^(*t*)^ is compactly written in a form of (*u*, *v*)^(*p*)^ where *u* = *w*_*i*_ and *v* = *w*_*i*__+__*l*_ are used during the sorting process to minimize the storage use. (*u*, *v*)^(*p*)^ is named *compact group p*. Any numeric data in between *u* and *v* are called *removed data*. These *removed data* are not considered and can be removed after the sorting process. For example, subsequence (1, 2, 3, 4, 5) is compacted as (1, 5)^(1)^; (4, 5, 7, 8, 10) is compacted as (4, 10)^(2)^; (5, 7, 8, 10) is compacted as (5, 10)^(3)^; and (8, 10, 12, 14) is compacted as (8, 14)^(4)^.

Note that a sequence *W*^(*t*)^ may contain several compact groups and some single numbers. Suppose *W*^(*t*)^ = (1, 2, 3, 4, 5, 7, 8, 10, 12, 14, 19). This sequence consists of the following subsequences (1, 5)^(1)^, (8, 14)^(4)^. Thus, *W*^(*t*)^ can be rewritten in another form of compact groups and a set of single numbers as *W*^(*t*)^ = ((1, 5)^(1)^, 7, (8, 14)^(4)^, 19). However, it is possible to find another set of compact groups from *W*^(*t*)^ as ${W^{(t)}}{ = (((1,3)^{(1)}}{,(4,7)^{(2)}}{,(8,14)^{(4)}},19)$. Obviously, different sets of compact groups for any *W*^(*t*)^ use different storage sizes to store them.

To distinguish between *W*^(*t*)^ written in the original sequence of numbers and *W*^(*t*)^ written in a form of compact groups having a set of single numbers, the notation *Q*^(*t*)^ is used instead of *W*^(*t*)^ to denote a combination set of compact groups and single numbers. Each compact group *i* in *Q*^(*t*)^ is denoted by *q*_*i*_. In fact, either each compact group or a single number in *Q*^(*t*)^ can be considered as an element of *Q*^(*t*)^. For example, if *W*^(*t*)^ = (1, 2, 3, 4, 5, 7, 8, 10, 12, 14, 19), then *Q*^(*t*)^ = ((1, 5)^(1)^, 7, (8, 14)^(4)^, 19) such that *q*_1_ = (1, 5)^(1)^, *q*_2_ = (8, 14)^(4)^. All *removed data* of compact group (*u*, *v*)^(*p*)^ will be occasionally retrieved to obtain a complete sorted subsequence in order to involve the new incoming subsequence in the sorting process. Hence, each retrieved number is denoted by *r*_*i*_ to make it different from each input number *w*_*i*_ during the sorting process. The retrieved sequence of (*u*, *v*)^(*p*)^, denoted *R*((*u*, *v*)^(*p*)^), can be obtained by using the following rules.

(2) $$r_{1} = u$$

(3) $$r_{1+l} = v$$

(4) $$r_{i+1} =\Bigg\{\matrix{r_{i}+1 & {{\rm for}\; p = 1} \cr r_{i}+(r_{i} - r_{1} + p-1)\; {\rm mod}\ 3 & {{\rm for}\; p = 2,3}\cr r_{i}+2 & {{\rm for}\; p = 4}}$$

To illustrate how to retrieve all numbers from a compact group, consider an example of sequence (5, 7, 8, 10) represented by the compact group (5, 10)^(3)^. The retrieved numbers of (5, 10)^(3)^ can be computed as follows: Since *p* = 3, *r*_1_ = 5, *r*_2_ = (5) + ((5) − (5) + (3) − 1) mod 3 = 7, *r*_3_ = (7) + ((7) − (5) + (3) − 1) mod 3 = 8, *r*_4_ = (8) + ((8) − (5) + (3) − 1) mod 3 = 10, *r*_4_ = 10 = *v*. Accordingly, $R\left( {{{(5,10)}^{(3)}}} \right) = (5,7,8,10)$.

## Concepts

The size of each incoming sequence is assumed to be at most the size of working storage. To make the working storage available for storing the next incoming data chunk after sorting the current chunk, it is required to represent some sorted subsequent numbers in a form of a *compact group*. However, not all subsequent sorted numbers can be compacted. The properties and concept of a compact group representation will be discussed next. The sorting process consists of the following three main steps.

1. Transform the initial incoming sequence *W*^(1)^ into a set of compact groups *q*_*i*_ ∈ *Q*^(1)^.
2. At time *t*, obtain the next incoming sequence and insert each number *w*_*i*_ ∈ *W*^(*t*)^ into the previous *Q*^(*t*^ ^− 1)^ at the appropriate position.
3. If there exist any adjacent compact groups *q*_*i*_ = (*a*,*b*)^(α)^ and *q*_*i*__+ 1_ = (*c*,*d*)^(β)^ such that the retrieved sequences *R*((*a*,*b*)^(α)^) and *R*((*c*,*d*)^(β)^) satisfy one of the types of subsequences, then form a new compact group from the sequences of *R*((*a*,*b*)^(α)^) and *R*((*c*,*d*)^(β)^).

**Figure 2:**
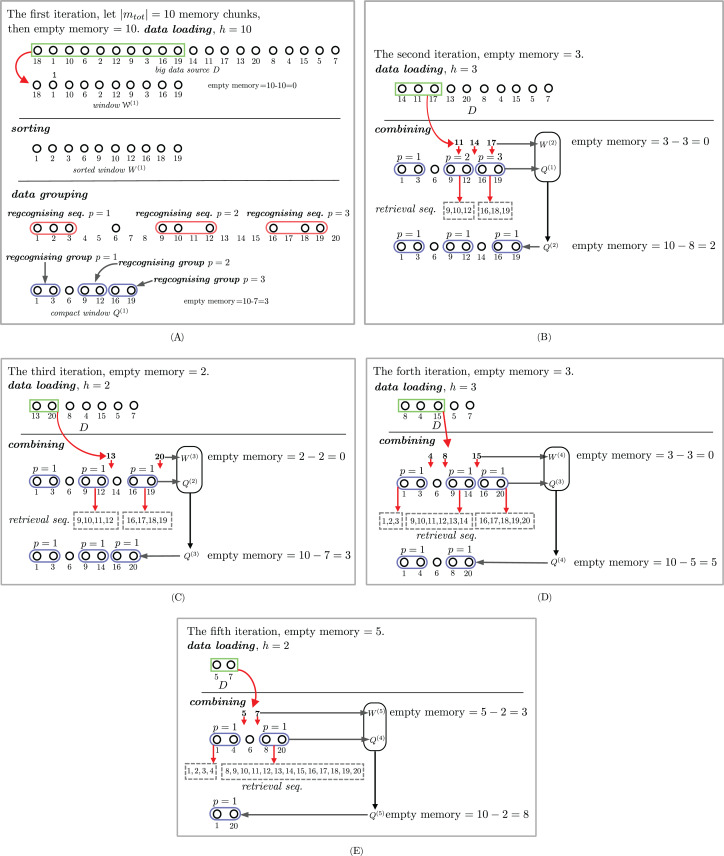
An example of streaming data sort. The sorting steps are illustrated in subfigures (A), (B), (C), (D) and (E).

Steps 2 and 3 are iterated until there are no more incoming sequences. The details of each step will be discussed next. Figure 2 shows an example of how the proposed approximate sorting works. The storage size |*m*_tot_| is 10. The first incoming 10-number sequence, that is, (18, 1, 10, 6, 2, 12, 9, 3, 16, 19), fills the whole storage. This sequence is sorted in an ascending order and forms a set *Q*^(1)^ = ((1, 3)^(1)^, 6, (9, 12)^(2)^, (16, 19)^(3)^), as shown in Fig. 2A. The size of the storage used is decreased to 7. The second incoming sequence (14, 11, 17) is inserted into some compact groups in *Q*^(1)^ to obtain *Q*^(2)^ = ((1, 3)^(1)^, 6, (9, 12)^(1)^, 14, (16, 19)^(1)^), as shown in Fig. 2B. The size of the storage used after the second incoming sequence is increased to 8. The third incoming sequence (13, 20) is separately grouped with (9, 12)^(1)^ and (16, 19)^(1)^ from the previous *Q*^(2)^ to make a new *Q*^(3)^ = ((1, 3)^(1)^, 6, (9, 13)^(1)^, 14, (16, 20)^(1)^). Observe that the compact group (9, 13)^(1)^ can be grouped with the single number 14 to make (9, 14)^(1)^. Therefore, *Q*^(3)^ = ((1, 3)^(1)^, 6, (9, 14)^(1)^, (16, 20)^(1)^). The fourth incoming sequence (8, 4, 15) is possibly and separately grouped with (9, 14)^(1)^, (1, 3)^(1)^, and (16, 19)^(1)^ in *Q*^(3)^ to obtain *Q*^(4)^ = ((1, 4)^(1)^, 6, (8, 20)^(1)^). The last incoming sequence (5, 7) is possibly and separately grouped with (1, 4)^(1)^, 6, (8, 20)^(1)^ in *Q*^(4)^ to obtain *Q*^(5)^ = ((1, 20)^(1)^).

## Proposed Algorithm

The proposed sorting algorithm is composed of the following two major steps. These steps are based on the constraints previously imposed in Constraints Section.

1. Obtain the first input number sequence and sort the number in an ascending order. Then, create *Q*^(1)^, a set of compact groups and a set of a single number.
2. At time *t*, obtain the next set of number sequences and insert the numbers into *Q*^(*t*^ ^− 1)^ to create the next *Q*^(*t*)^.
3. Repeat step 2 until there are no more new incoming sequences.

The deils of steps 1 and 2 will be discussed in the following sections.

### Creating compact groups

There are four types of compact groups. To identify the type of compact group from a number sequence, four counters *c*_1_, *c*_2_, *c*_3_, and *c*_4_ for type-1, type-2, type-3, and type-4, respectively, are employed. Let *s*^(*i*)^ be the status condition of type-*i*. The value of *s*^(*i*)^ is defined as follows.

**Definition 7** *Type-1 status condition s*^(1)^ *of a datum w*_*k + i*_ *and its neighbors in a type-1 subsequence w*_*k*_*,…,w*_*k + i*_*,…,w*_*k + m*_, *where m < h is a constant defined by:*

$${s^{(1)}} = \left\{ {\matrix{ 1 & {{w_{k + i}} - {w_{k + i - 1}} = 1\;\;{\rm for}\;\;0 \le i \le m} \cr 0 & {{\rm otherwise}{\rm .}} \cr } } \right.$$

**Definition 8** *Type-2 status condition s*^(2)^ *of a datum w*_*k + i*_ *and its neighbors in a type-2 subsequence w*_*k*_*,…,w*_*k + i*_*,…,w*_*k + m*_, *where m < h is a constant defined by:*

$${s^{(2)}} = \left\{ {\matrix{ 1 & {({w_{k + i - 1}} - {w_k})\bmod 3 + 1 = {w_{k + i}} - {w_{k + i - 1}}\;\;{\rm for}\;\;0 \le i \le m} \cr 0 & {{\rm otherwise}{\rm .}} \cr } } \right.$$

**Definition 9** *Type-3 status condition s*^(3)^ *of a datum w*_*k + i*_ *and its neighbors in a type-3 subsequence w*_*k*_*,…,w*_*k + i*_*,…,w*_*k + m*_, *where m<h is a constant defined by:*

$${s^{(3)}} = \left\{ {\matrix{ 1 & {({w_{k + i - 1}} - {w_k} + 2)\bmod 3 = {w_{k + i}} - {w_{k + i - 1}}\;\;{\rm for}\;\;0 \le i \le m} \cr 0 & {{\rm otherwise}{\rm .}} \cr } } \right.$$

**Definition 10** *Type-4 status condition s*^(4)^ *of a datum w*_*k + i*_ *and its neighbors in a type-4 subsequence w*_*k*_*,…,w*_*k + i*_*,…,w*_*k + m*_, *where m < h is a constant defined by:*

$${s^{(4)}} = \left\{ {\matrix{ 1 & {{w_{k + i}} - {w_{k + i - 1}} = 2\;\;{\rm for}\;\;0 \le i \le m} \cr 0 & {{\rm otherwise}{\rm .}} \cr } } \right.$$

The notations in this paper are given in Table 2.

**Table 2:** Notations in streaming data sort.

Notations	Short definitions	Examples
*d*_i_	The *i*^*th*^ incoming datum	−3, 0, 10
(*d*_1_,*d*_2_,*d*_3_,…)	Sequence of streaming data	(−3, 0, 10,…)
*h*	Window size at iteration *t*	5, 0, 4, 1
*w*_i_	The *i*^*th*^ member in a window	18, 1, 10
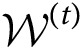	Unsorted window at iteration *t*	(18, 1, 10, 6,…)
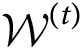	Sorted window at iteration *t*	(1, 6, 10, 18,…)
*p*	Type of sub-sequence	1, 2, 3, 4
*T*_*p*_	Type-*p* sub-sequence	(2, 4, 6, 8, 10, 12)
(*u*, *v*)^*(p)*^	Type-*p* compact group	(2, 12)^(3)^
*r*_*i*_	The *i*^*th*^ retrieved number	2, 4, 6, 8, 10, 12
*R*((*u*, *v*)^*(p)*^)	Retrieved sequence of (*u*, *v*)^*(p)*^	(2, 4, 6, 8, 10, 12)
*s*^*(i)*^	Status condition of type-*i*	0, 1
*q*_*i*_	The *i*^*th*^ compact group	(1, 5)^(4)^, (9, 12)^(1)^
*S*	Set of single numbers	{6, 7}
*C*	Set of compact groups	{(1, 5)^(4)^, (9, 12)^(1)^}
*Q*^*(t)*^	Combining set of *C* and *S* at iteration *t*	{(1, 5)^(4)^, 6, 7, (9, 12)^(1)^}

*Q*^(*t*)^||*S*|| *C* denotes orderly concatenating *Q*(*t*), *S*, *C* according to the sorted order of all elements in *W*^(*t*)^. The quantity of removed data of type-1 is greater than those of the other types. The difference of the first and the last data of a type-4 compact group is larger than the differences in the other types. To greatly reduce and control the storage size, the sequences of types 1 and 4 are detected before the sequences of types 2 and 3. Suppose the following sequence (1, 3, 4, 5, 6) is given. If types 1 and 4 are considered before types 2 and 3, then the given sequence is compacted as 1, (3, 6)^(1)^, which requires 4 units of storage to store numbers 1, 3, 6, 1. However, if types 2 and 3 are considered before types 1 and 4, then the given sequence is compacted as (1, 4)^(2)^, 5, 6, which requires 5 units of storage to store numbers 1, 4, 2, 5, 6.

**Theorem 1** *If* $p = {\rm arg}\,\,\mathop {\max }\limits_{1 \le i \le 4} ({c_i})$, *then p denotes the correct type of the compact group*.

**Proof:** Suppose the sorted sequence is *W*^(*t*)^ = (*w*_1_, *w*_2_, …, *w*_*h*_). We consider each type of compact group. Let *s*^(*i*)^_*t*_ be the status condition of type-*i* at time *t* and *T*^(*i*)^ = (*s*^(*i*)^_1_, *s*^(*i*)^_2_, …, *s*^(*i*)^_*h*_) be the sequence of *s*^(*i*)^_*t*_. There are four cases to be investigated.

*Case 1 (type-1):* Suppose the sorted sequence *W*^(*t*)^ = (*w*_1_, *w*_2_, …, *w*_*h*_) is in type-1. Then, we have the following four sequences of the status condition.

$$\eqalign{T^{(1)} = \left(s^{(1)}_{1} = 0, s^{(1)}_{2} = 0, s^{(1)}_{3} = 1,\ldots,s^{(1)}_{h} = 1\right) {\ {\rm or}\ (0, 0, 1,\ldots, 1)} \cr T^{(2)} = \left(s^{(2)}_{1} = 0, s^{(2)}_{2} = 0, s^{(2)}_{3} = 0,\ldots,s^{(2)}_{h} = 0\right) {\ {\rm or}\ (0, 0, 0,\ldots, 0)} \cr T^{(3)} = \left(s^{(3)}_{1} = 0, s^{(3)}_{2} = 0, s^{(3)}_{3} = 0,\ldots,s^{(3)}_{h} = 0\right) {\ {\rm or}\ (0, 0, 0,\ldots, 0)} \cr T^{(4)} = \left(s^{(4)}_{1} = 0, s^{(4)}_{2} = 0, s^{(4)}_{3} = 0,\ldots,s^{(4)}_{h} = 0\right) {\ {\rm or}\ (0, 0, 0,\ldots, 0)}} $$

Obviously, the value of ${c_1} = \sum\nolimits_{t = 1}^h s_t^{(1)}$ is larger than that of *c*_2_, *c*_3_, and *c*_4_.

*Case 2 (type-2):* Suppose the sorted sequence *W*^(*t*)^ = (*w*_1_, *w*_2_, …, *w*_*h*_) is in type-2. Then, we have the following four sequences of the status condition.

$$\eqalign{T^{(1)} = \left(s^{(1)}_{1} = 0, s^{(1)}_{2} = 0, s^{(1)}_{3} = 0,\ldots,s^{(1)}_{h} = 0\right) {\ {\rm or}\ (0, 0, 0,\ldots, 0)} \cr T^{(2)} = \left(s^{(2)}_{1} = 0, s^{(2)}_{2} = 0, s^{(2)}_{3} = 1,\ldots,s^{(2)}_{h} = 1\right) {\ {\rm or}\ (0, 0, 1,\ldots, 1)} \cr T^{(3)} = \left(s^{(3)}_{1} = 0, s^{(3)}_{2} = 0, s^{(3)}_{3} = 0,\ldots,s^{(3)}_{h} = 0\right) {\ {\rm or}\ (0, 0, 0,\ldots, 0)} \nonumber \cr T^{(4)} = \left(s^{(4)}_{1} = 0, s^{(4)}_{2} = 0, s^{(4)}_{3} = 0,\ldots,s^{(4)}_{h} = 0\right)\ {{\rm or}\ (0, 0, 0,\ldots, 0)}} $$

Obviously, the value of ${c_2} = \sum\nolimits_{t = 1}^h s_t^{(2)}$ is larger than that of *c*_1_, *c*_3_, and *c*_4_.

*Case 3 (type-3):* Suppose the sorted sequence *W*^(*t*)^ = (*w*_1_, *w*_2_, …, *w*_*h*_) is in type-3. Then, we have the following four sequences of the status condition.

$$\eqalign{T^{(1)} = \left(s^{(1)}_{1} = 0, s^{(1)}_{2} = 0, s^{(1)}_{3} = 0,\ldots,s^{(1)}_{h} = 0\right) {\ {\rm or}\ (0, 0, 0,\ldots, 0)} \cr T^{(2)} = \left(s^{(2)}_{1} = 0, s^{(2)}_{2} = 0, s^{(2)}_{3} = 0,\ldots,s^{(2)}_{h} = 0\right) {\ {\rm or}\ (0, 0, 0,\ldots, 0)} \cr T^{(3)} = \left(s^{(3)}_{1} = 0, s^{(3)}_{2} = 0, s^{(3)}_{3} = 1,\ldots,s^{(3)}_{h} = 1\right) {\ {\rm or}\ (0, 0, 1,\ldots, 1)} \cr T^{(4)} = \left(s^{(4)}_{1} = 0, s^{(4)}_{2} = 0, s^{(4)}_{3} = 0,\ldots,s^{(4)}_{h} = 0\right) {\ {\rm or}\ (0, 0, 0,\ldots, 0)}} $$

Obviously, the value of ${c_3} = \sum\nolimits_{t = 1}^h s_t^{(3)}$ is larger than that of *c*_1_, *c*_2_, and *c*_4_.

*Case 4 (type-4):* Suppose the sorted sequence *W*^(*t*)^ = (*w*_1_, *w*_2_, …, *w*_*h*_) is in type-4. Then, we have the following four sequences of the status condition.

$$\eqalign{T^{(1)} = \left(s^{(1)}_{1} = 0, s^{(1)}_{2} = 0, s^{(1)}_{3} = 0,\ldots,s^{(1)}_{h} = 0\right) {\ {\rm or}\ (0, 0, 0,\ldots, 0)} \cr T^{(2)} = \left(s^{(2)}_{1} = 0, s^{(2)}_{2} = 0, s^{(2)}_{3} = 0,\ldots,s^{(2)}_{h} = 0\right) {\ {\rm or}\ (0, 0, 0,\ldots, 0)} \cr T^{(3)} = \left(s^{(3)}_{1} = 0, s^{(3)}_{2} = 2, s^{(3)}_{3} = 0,\ldots,s^{(3)}_{h} = 0\right) {\ {\rm or}\ (0, 0, 0,\ldots, 0)} \cr T^{(4)} = \left(s^{(4)}_{1} = 0, s^{(4)}_{2} = 0, s^{(4)}_{3} = 1,\ldots,s^{(4)}_{h} = 1\right) {\ {\rm or}\ (0, 0, 1,\ldots, 1)}} $$

Obviously, the value of ${c_4} = \sum\nolimits_{t = 1}^h s_t^{(4)}$ is larger than that of *c*_1_, *c*_2_, and *c*_3_.▪

### Inserting numbers into the combination set of compact groups

After creating the first combination set of compact group *Q*^(1)^ and obtaining a new incoming sequence, the current compact groups must be updated according to the number in the incoming sequence. There are seven possible cases where a new incoming number can be inserted into any compact group or in between a compact group and a single number. Let the α new incoming number *d*_α_ is located according to each case as follows. *Q*^(*t*)^ is the set of combinations of compact groups and a set of single numbers at time *t*.

*Case 1*: *d*_α_ is at the front of *Q*^(*t*)^. *Case 2*: *d*_α_ is at the rear of *Q*^(*t*)^. *Case 3*: *d*_α_ is in a compact group (*u*_*j*_, *v*_*j*_)^(*p*)^. *Case 4*: *d*_α_ is in between two compact groups (*u*_*j*_, *v*_*j*_)^(^^*p_j_*^^)^ and (*u*_*k*_, *v*_*k*_)^(*p*_*k*_^^)^. *Case 5*: *d*_α_ is in between a single number *w*_*j*_ and a compact group (*u*_*k*_, *v*_*k*_)^(*p*_*k*_^^)^. *Case 6*: *d*_α_ is in between a compact group (*u*_*j*_, *v*_*j*_)^(*p*_*j*_^^)^ and a single number *w*_*k*_. *Case 7*: *d*_α_ is in between two single numbers *w*_*j*_ and *w*_*j*__+ 1_.

The details of each case and the insertion steps are in given in Algorithm 2.

## Experimental Results and Discussion

Three issues are discussed in this section. The first issue illustrates the snapshot of sorting outcomes as the results of incoming data chunks, current compact groups of different types, and sets of single numbers. The second issue discusses the relation between the sorting time and the number of streaming numbers. The third issue shows how the size of working storage changes during the sorting process.

### Sorting examples

The proposed algorithms are implemented in MATLAB R2016a. The computing results are run on 3.4 GHz Intel Core i7 6700 and 16 GB of 2400 MHz RAM with the Windows 10 platform. To illustrate how the proposed algorithm works, three experiments were conducted by using a set of 100 single integers ranging from 1 to 100. These 100 numbers were randomly permuted to produce three different experimental data sets. The total size of storage is assumed to have only 60 working addresses. Forty of them are used for storing temporary data generated during the sorting process, which includes 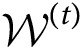, *W*^(*t*)^, and *Q*^(*t*)^ at different times. The rest of storage is for storing some variables in the sorting program.

**Figure 3:**
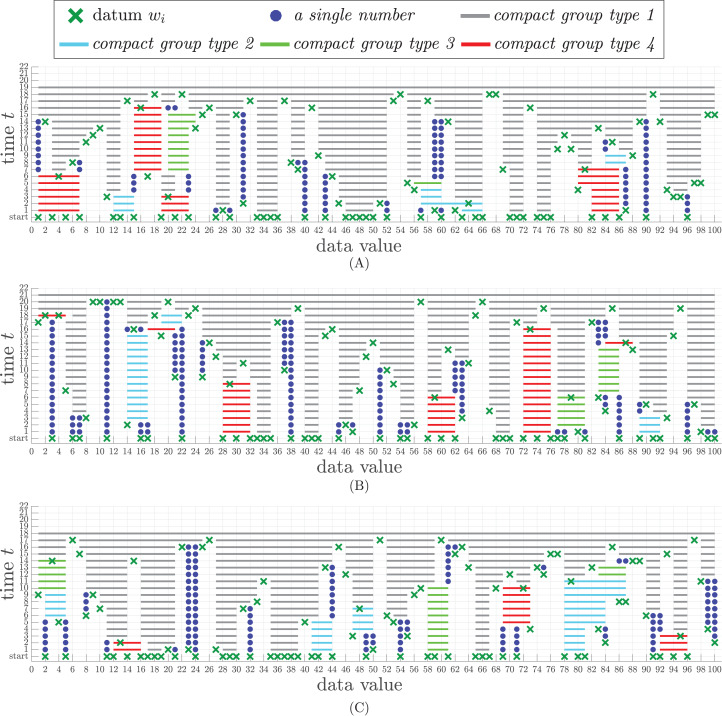
Snapshots of sorting results from three different permuted data sets, each of which contains 100 numbers. (A) The time steps of the sorting result of data set 1. (B) The time steps of the sorting result of data set 2. (C) The time steps of the sorting result of data set 3.

To illustrate the continuous results during the sorting process, three data sets in the experiment were generated from three permutations of integer numbers from 1 to 100 to avoid any duplication. These permuted numbers are sliced into a set of input chunks of at most 40 numbers in each chunk. Let $\left| {{m_{tot}}} \right|$ be the total size of the working storage, which is equal to 60 in the experiment. The experimental results are shown in Fig. 3, where the x-axis represents each *w*_*i*_ in *W*^(*t*)^ and *Q*^(*t*)^ and the *y*-axis represents the time line of iterations. Each datum *w*_*i*_ is represented by ×. Each type of compact group in *Q*^(*t*)^ is denoted by a solid line with a specific color as follows.

Type-1 is denoted by gray line.
Type-2 is denoted by blue line.
Type-3 is denoted by green line.
Type-4 is denoted by red line.

A single number in the compact window is represented by •.

In the first data set, there are 40 numbers entering the process at the starting time. After being grouped by Algorithm 1, the result appears in time step *t* = 1 (at the line above the bottom line in Fig. 3A. There are four compact groups of type-1, two compact groups of type-2, three compact groups of type-4, and nine single numbers. Also, at time *t* = 1, there are four new incoming numbers, each of which is represented by ×. Algorithm 1 sorts all incoming 100 numbers appearing in various chunks within only 19 time steps, whereas it takes 21 and 18 time steps for the second and the last data sets, respectively.

**Algorithm 1:** Creating compact groups.

**Input:** a sorted sequence *W*^*(t)*^ = (*w_k_*, *w_k_*_+1_, …, *w_k_*_+_*_h_*) of length *h* at time *t*.
**Output:** a combination of a set of compact groups and a set of single numbers.
1. *j* = *k*.
2. *S* = Ø. /* set of single numbers */
3. *C* = Ø. /* set of compact groups */
4. *Q*^(*t*)^ = Ø.
5. **For** *l* = 1 **to** 2 **do** /* packing order types 1, 4 before 2, 3 */
6. *c*_1_ = *c*_2_ = *c*_3_ = *c*_4_ = 0.
7. **If** |*w_k_* −*w_k_*_+1_| > 2 **then**
8. *S* = *S*∪{*w_k_*}.
9. *j* = *k*+1.
10. **EndIf**
11. **For** *i* = *k*+1 **to** *k*+*h*−1 **do**
12. **If** |*w_i_*−*w_i_*_+1_| ≤ 2 **then**
13. Set the values of *s*^(1)^, *s*^(2)^, *s*^(3)^, *s*^(4)^ by definitions 7–10.
14. *c_l_* = *c_l_* +*s*^(*l*)^.
15. *c*_5−*l*_ = *c*_5−*l*_ +*s*^(5−*l*)^.
16. **else**
17. **If** *j* = *i* **then**
18. *S* = *S*∪{*w_j_*}. /* single number */
19. *j* = *i* +1.
20. **else**
21. *p* = *arg* $\max_{i\in\{{l,5-l\}}}$ (*c_i_*). /* compact types */
22. Create a compact group (*w_j_*, *w_i_*)^(*p*)^.
23. *C* = *C*∪{(*w_j_*, *w_i_*)^(*p*)^}.
24. *c*_1_ = *c*_2_ = *c*_3_ = *c*_4_ = 0.
25. *j* = *i* + 1.
26. **EndIf**
27. **EndIf**
28. *Q*^(*t*)^ = *Q*^(*t*)^||*S*||*C*.
29. **EndFor**
30. **EndFor**

### Execution time vs working storage size

In this experiment, the relation between the total numbers to be sorted and the sorting time was investigated. The total storage, *m*_tot_, is partitioned into two portions. The first portion, *m*_prog_, is for the sorting program. The size of *m*_prog_ is fixed throughout the sorting process. The second portion, *m*_work_, is the working storage for storing all compact groups, sets of single numbers, and other relevant variables occurring during in the sorting algorithm. Since the sorting time directly depends upon the size of *m*_work_, the size of *m*_work_ is thus set as a function of the total numbers to be sorted. Let *n* ≫|*m*_work_| be the total numbers to be sorted. All numbers to be sorted flow gradually and continuously into the working storage one chunk at an initial time. To investigate the execution time of the sorting process with respect to the quantity of numbers and |*m*_work_|, the size of *m*_work_ is set in terms of *n* as follows.

(5) $$|{m_{\rm work}}| = {\rm{\gamma}} \times n$$

where γ ∈ {0.50, 0.45, 0.40, 0.35} and *n* ∈ {10^3^, 10^4^, 10^5^, 10^6^}.

**Figure 4:**
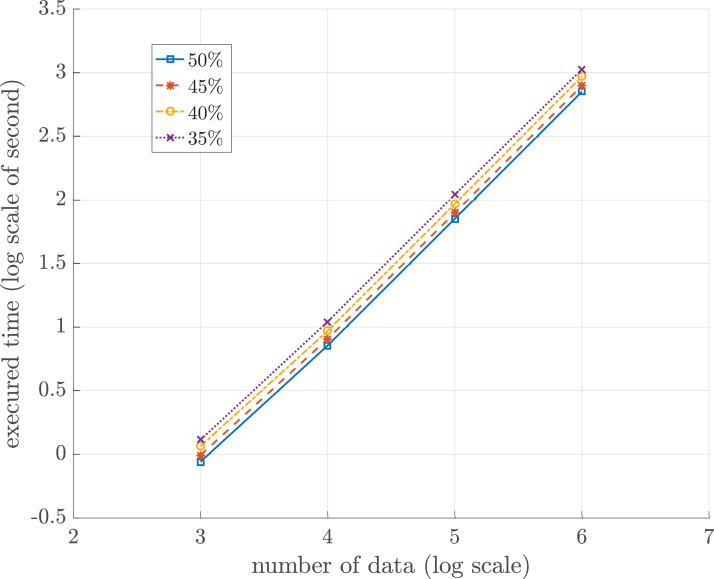
Log-scaled sorting execution time in seconds for different sizes of working storage *n* = 10^3^, 10^4^, 10^5^ and 10^6^.

Table 3 summarizes the proposed sorting algorithm time of different quantities of incoming numbers with respect to the different sizes of the working memory. The incoming numbers were randomly generated and permuted. No duplicated numbers appear in the data sets. To visualize the trend of the sorting time vs the size of data sets, Fig. 4 shows the log-scaled trend of each data set. There are four lines in blue, red, yellow, and purple representing different sizes of *m*_work_. Note that the sorting time of each data set linearly increases. Then, the experiment has a linear polynomial time complexity of *O*(*n*). Table 4 summarizes the time of *external sorting* of different quantities of incoming numbers with respect to the different sizes of buffer. The execution time of proposed sorting algorithm is approximately 8.72 times faster than the execution time of *external sorting* at a million data when storage size is limited to 50%. Furthermore, the proposed algorithm run on 4 GB of numeric data takes about 4.21 days.

**Table 3:** Sorting execution time of the proposed algorithm with respect to size of working storage.

*n*	Execution time (s)			
	γ = 50%	γ = 45%	γ = 40%	γ = 35%
10^3^	0.87	0.98	1.16	1.30
10^4^	7.15	8.08	9.33	11.02
10^5^	71.06	79.17	92.80	109.88
10^6^	709.49	791.17	933.80	1,065.71

**Table 4:** Sorting execution time of external sorting with respect to buffer size.

*n*	Execution time (s)			
	Buffer = 50%	Buffer = 45%	Buffer = 40%	Buffer = 35%
10^3^	2.30	2.69	2.64	2.66
10^4^	26.95	21.04	20.19	20.18
10^5^	209.41	207.27	206.87	205.15
10^6^	6,187.14	5,312.09	4,754.30	4,539.04

### Fluctuation of compact groups and single number sets

**Figure 5:**
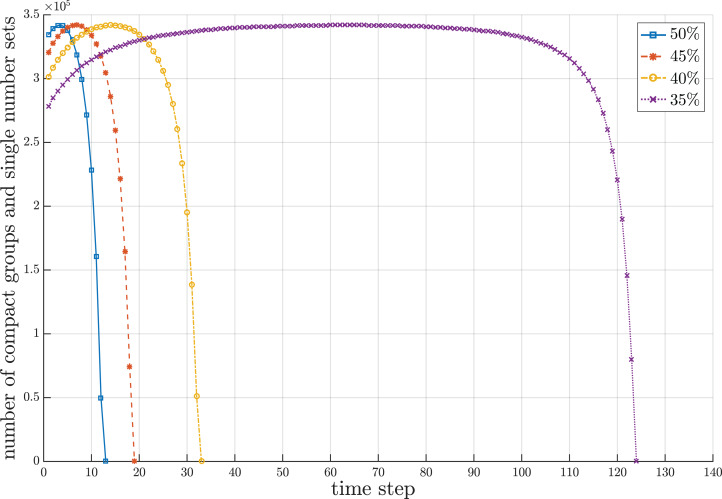
Snapshot of fluctuation of compact groups and sets of single numbers at a million data during the sorting period for different sizes of working storage.

**Figure 6:**
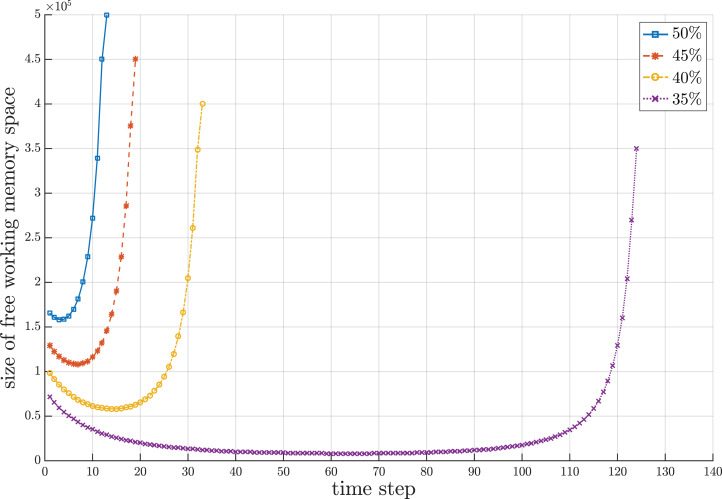
Snapshot of fluctuation of unused area of working storage at a million data during the sorting period for different sizes of working storage.

Since the proposed sorting algorithm is designed to cope with a streaming data environment where the set of numbers to be sorted can overflow the working storage and the chunks of numbers gradually flow into the working storage, there are three interesting behavioral periods concerning the number of compact groups and sets of single numbers created during the sorting process. It is remarkable that the number of compact groups and sets of single numbers increase during the beginning period due to random values of incoming numbers. The length of beginning period depends upon the random sequence of numbers, which is unpredictable. After the beginning period, some new incoming numbers may fall into the existing compact groups and some of them may form new compact groups with some sets of single numbers. Some existing compact groups can be merged with new compact groups created from some sets of single numbers into new compact groups. These conditions make the number of compact groups almost stable for some period of time. In the last period, those new incoming numbers obviously fall to combine with the existing compact groups. Some sequences of compact groups are possibly merged into new compact groups with more elements in the groups. Thus, the number of compact groups decreases until there is one compact group that contains all sorted numbers. Figure 5 illustrates the fluctuation of compact groups with sets of single numbers vs the time steps for different sizes of working storage. During the sorting process, the number of compact groups and sets of single numbers increases and decreases. The fluctuation of used and unused areas of working storage of the results in Fig. 5 is summarized in Fig. 6. Notice that the proposed algorithm can reduce the working space to 65% of the data size. In the other words, the working space of the proposed algorithm is 35% of the data size.

### Comparison of storage size used and correctness of sorted order

**Figure 7:**
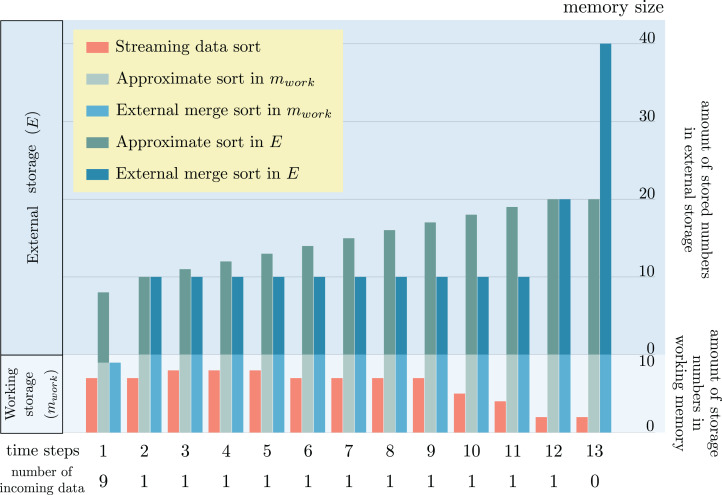
Comparison of storage size change as the results of sorting a set of streaming data by the proposed streaming data sort algorithm, approximate sorting (Farnoud, Yaakobi & Bruck, 2016) algorithm and external sorting.

Regardless of the sorting types, either exact sort or approximate sort, the order of each number in the sorted list must be correct according to the value of each number for both ascending and descending sorts. If it is not so, the sorted list is useless in any applications. To verify the efficiency and the accurate order of sorted numbers as the result of proposed *streaming data sort* in a streaming data environment with limited storage size, the result was compared with the result of *approximate sorting* algorithm (Farnoud, Yaakobi & Bruck, 2016) capable of handling streaming data, and *external sorting*. The following set of numbers was experimented: {18, 1, 10, 6, 2, 12, 9, 3, 16, 19, 14, 11, 17, 13, 20, 8, 4, 15, 5, 7}. In order to simulate streaming data, the set of numbers was decomposed into several consecutive chunks. The first incoming chunk contains nine numbers. The other following chunks contain only one number. Two issues concerning the change of storage size during the sorting process and the wrong sorted order were recorded in the experiment. Since *streaming data sort* algorithm uses only one working storage of fixed size throughout the sorting process, there is no change of storage size for this algorithm. But in case of *approximate sorting* and *external sorting* algorithms, both of them require working storage of fixed size and also external storage of variable size. Hence, the change of storage size only occurs in the external storage. Figure 7 snapshots the storage size change at each time. *m*_work_ is a constant denoting the fixed size of working storage. The size of external storage is expandable according to the amount of temporary data generated during the sorting algorithms.

It is remarkable that the proposed *streaming data sort* does not require any space in the external storage. Only working storage space alone is enough to complete the sorting process. But both *approximate sorting* and *external sorting* need some additional spaces in the external storage. These spaces keep increasing when there are more incoming numbers to be sorted. The sorted order of all numbers obtained from *streaming data sort* is perfectly correct. But the sorted orders of numbers 7, 8, 13, 14 obtained from *approximate sorting* are not correct. Although the sorted order obtained from *external sorting* is perfectly correct, this algorithm requires a large size of external storage which is impractical for streaming data environment.

### Time complexity analysis

There are two main phases in the sorting process. The first phase is to sort the first incoming chunk of numbers to obtain the first set of compact groups as well as sets of single numbers. The second phase is to sort the consequent chunks with the existing compact groups and single numbers. Let *h* ≤ |*m*_work_| be the size of each input chunk. The time complexity of each phase is as follows.

*Phase 1:* The operation of this phase is in Algorithm 1. Obtaining *h* numbers takes *O*(*h*). These *h* numbers must be sorted to create compact groups and sets of single numbers. The time to sort *h* numbers is *O*(*h* log (*h*)). After sorting, the time to create compact groups and sets of single numbers takes *O*(*h*). Thus, the time of this phase is *O*(*h*) + *O*(*h* log(*h*)) + *O*(*h*) = *O*(*h* log(*h*)).

*Phase 2:* From Algorithm 1, all compact groups at any time are in set *C*, and all single numbers are in set *S*. The time complexity of this phase can be analyzed from Algorithm 2. There are seven cases to be identified for inserting a new number *d*_α_ at step 1. The identifying time takes *O*(|*C*|) + *O*(|*S*|) $= \max (O(|C|),O(|S|))$. Then, applying Eqs. (2), (3) and (4) to retrieve the numbers from a compact group takes *O*(1). After retrieval of the numbers, Algorithm 1 is applied to create a new compact group and a set of single numbers with the new incoming *d*_α_. This step takes at most *O*(*h*). At steps 32–34, Algorithm 1 is repeatedly applied to update sets *C* and *S*. This takes at most *O*(*h*×|*C*|) + *O*(|*S*|). Since |*C*| ≤ *h* and |*S*| ≤ *h*, the time complexity of steps 32–34 is *O*(*h*^2^). Thus, phase 2 takes max(*O*(|*C*|), *O*(|*S*|)) + *O*(1) + *O*(*h*) + *O*(*h*^2^) = *O*(*h*^2^) for each *d*_α_. If there are in total *n* numbers to be sorted, then the time complexity is *O*(*h* log(*h*)) + *O*((*n* − *h*) × *h*^2^) = *O*(*nh*^2^). However, *h* is a constant. Hence, the time complexity of the sorting process is *O*(*n*).

**Algorithm 2:** Inserting *d*_α_ into *Q*(*t*).

**Input:** (1) set *Q*^(*t*)^. (2) a new number *d*_α_.
**Output:** a new *Q*^(*t*+1)^.
1. Identify the case of insertion for *d*_α_.
2. **Case:**
3. 1: **If** the first element of *Q*^(*t*)^ is (*u*_1_, *v*_1_)^(*p*_1_^^)^
**then**
4. Let *U* be the retrieved (*u*_1_, *v*_1_)^(*p*_1_^^)^ by using Eqs. (2), (3) and (4).
5. **else**
6. Put *d*_1_ in *U*.
7. **EndIf**
8. Use Algorithm 1 with *d*_α_ and *U* to generate a new set of elements.
9. **EndCase**
10. 2: **If** the last element of *Q*^(*t*)^ is (*u_m_*, *v_m_*)^(*p_m_*^^)^
**then**
11. Let *U* be the retrieved (*u_m_*, *v_m_*)^(*p_m_*^^)^ by using Eqs. (2), (3) and (4).
12. **else**
13. Put *d_m_* in *U*.
14. **EndIf**
15. Use Algorithm 1 with *d*_α_ and *U* to generate a new set of elements.
16. **EndCase**
17. 3: Let *U* be the retrieved (*u_m_*, *v_m_*)^(*p_m_*^^)^ by using Eqs. (2), (3) and (4).
18. Use Algorithm 1 with *d*_α_ and *U* to generate a new set of elements.
19. **EndCase**
20. 4: Let *U* be the retrieved (*u_j_*, *v_j_*)^(*p_j_*^^)^ by using Eqs. (2), (3) and (4).
21. Let *V* be the retrieved (*u_k_*, *v_k_*)^(*p_k_*^^)^ by using Eqs. (2), (3) and (4).
22. Use Algorithm 1 with *d*_α_, *U* and *V* to generate a new set of elements.
23. **EndCase**
24. 5: Let *U* be the retrieved (*u_k_*, *v_k_*)^(*p*_*k*_^^)^ by using Eqs. (2), (3) and (4).
25. Use Algorithm 1 with *d*_α_, *w_j_* and *U* to generate a new set of elements.
26. **EndCase**
27. 6: Let *U* be the retrieved (*u_j_*, *v_j_*)^(*p_j_*^^)^ by using Eqs. (2), (3) and (4).
28. Use Algorithm 1 with *d*_α_, *U* and *w_k_* to generate a new set of elements.
29. **EndCase**
30. 7: Use Algorithm 1 with *d*_α_, *w_j_* and *w_k_* to generate a new set of elements.
31. **EndCase**
32. **Repeat**
33. Use Algorithm 1 with the new set of elements and the unpacked element next to the new set next to the new set of elements to generate the next new set of elements in *Q*^(*t*)^.
34. **Until** no more new elements.
35. Rename *Q*^(*t*)^ as *Q*^(*t*+1)^.
36. **EndCase**

### Storage usage analysis

The behavior of storage usage is in the form of a capsized bell shape, as shown in Fig. 5. The descriptive rationale behind this behavior was briefly provided in Fluctuation of Compact Groups and Single Number Sets Section. This section will theoretically analyze this behavior based on the probability of all seven cases for a compact group. Suppose there are *n* total streaming numbers to be sorted. All incoming *n* numbers are assumed to be randomly permuted and partitioned into ${n \over h}$ input chunks of size *h* each. Let *n*_*i*_ be the numbers in the *i^th^* input data chunk. After obtaining the first input data chunk, the probability of each case for the next new incoming number *d*_α_ for any compact group *q*_*i*_ is as follows.

*Case 1*: *d*_α_ is at the front of *q*_*i*_. The probability of case 1 is calculated by the probability of picking *d*_α_ from *n* − *n*_1_ and the probability of having *d*_α_ in the next input chunk. The probability of picking *d*_α_ from *n* − *n*_1_ numbers is ${1 \over {n - {n_1}}}$. However, if *d*_α_ is the next new incoming number, then *d*_α_ must be in the next input data chunk. The probability that *d*_α_ is in the next input chunk is ${{{1 \over n}} \over {h - 1}}$. Thus, the probability of case 1 is as follows.

(6) $${p_1} = \displaystyle{1 \over {n - {n_1}}} \cdot \displaystyle{1 \over {{n \over h} - 1}}$$

*Case 2*: *d*_α_ is at the rear of *q*_*i*_. The probability of case 2 can be analyzed as that of case 1.

*Case 3*: *d*_α_ is in a compact group *q*_*i*_, types 2, 3, and 4 are compact groups only.

If *q*_*i*_ is a type-2 compact group, then the probability that *d*_α_ is in *q*_*i*_ is $\big\lfloor {{{|{q_i}|} \over 2}} \big\rfloor \cdot {1 \over {n - {n_1}}}$, where |*q*_*i*_| represents the numbers compacted in *q*_*i*_.

If *q*_*i*_ is a type-3 compact group, then the probability that *d*_α_ is in *q*_*i*_ is $\big\lfloor {{{|{q_i}|} \over 2}} \big\rfloor \cdot{1 \over {n - {n_1}}}$.

If *q*_*i*_ is a type-4 compact group, then the probability that *d*_α_ is in *q*_*i*_ is ${{\left( {|{q_i}| - 1} \right)} \over {n - {n_1}}}$.

(7) $${p_3} = \left\{ {\matrix{ {\bigg\lfloor {\displaystyle{{|{q_i}|} \over 2}} \bigg\rfloor \cdot \displaystyle{1 \over {n - {n_1}}} \cdot \displaystyle{1 \over {{n \over h} - 1}}} & {{\rm types}\;{\rm 2}\;{\rm or}\;{\rm 3}} \cr {\displaystyle{{|{q_i}| - 1} \over {n - {n_1}}} \cdot \displaystyle{1 \over {{n \over h} - 1}}} & {{\rm type}\;{\rm 4}{\rm .}} \cr } } \right.$$

*Case 4*: *d*_α_ is in between two compact groups *q*_*i*_ and *q*_*i*__+ 1_. The probability of case 4 can be analyzed as that of case 1.

*Case 5*: *d*_α_ is in between a single number *w*_*j*_ and a compact group *q*_*i*_.

If *q*_*i*_ is a type-1 compact group, then the probability that *d*_α_ is in *q*_*i*_ is ${1 \over {n - {n_1}}}$.

If *q*_*i*_ is a type-2 compact group, then the probability that *d*_α_ is in *q*_*i*_ is ${1 \over {n - {n_1}}}$.

If *q*_*i*_ is a type-3 compact group, then the probability that *d*_α_ is in *q*_*i*_ is ${1 \over {n - {n_1}}}$.

If *q*_*i*_ is a type-4 compact group, then the probability that *d*_α_ is in *q*_*i*_ is ${1 \over {n - {n_1}}}$.

The probability of case 5 can be analyzed as that of case 1.

*Case 6*: *d*_α_ is in between a compact group *q*_*i*_ and a single number *w*_*k*_. The probability of case 6 can be analyzed as that of case 1.

*Case 7*: *d*_α_ is in between two single numbers *w*_*j*_ and *w*_*j*__+ 1_. The probability of case 7 can be analyzed as that of case 1.

Note that the probability of all cases for the first input data chunk is written as follows.

(8) $$p = \left\{ {\matrix{ {\left\lfloor {\displaystyle{{|{q_i}|} \over 2}} \right\rfloor \cdot \displaystyle{1 \over {n - {n_1}}} \cdot \displaystyle{1 \over {{n \over h} - 1}}} & \text{case 3 (type 2 or type 3)} \cr\hskip-2pc {\displaystyle{{|{q_i}| - 1} \over {n - {n_1}}} \cdot \displaystyle{1 \over {{n \over h} - 1}}} & \hskip-3.4pc\text{case 3 (type 4)} \cr\hskip-2.1pc {\displaystyle{1 \over {n - {n_1}}} \cdot \displaystyle{1 \over {{n \over h} - 1}}} & \hskip-4.8pc{\rm other\ cases} \cr } } \right.$$

After the first input data chunk, the probability of each case after *m* next input data chunks can be written in a generic form as follows.

(9) $$p = \left\{ {\matrix{ {\left\lfloor {\displaystyle{{|{q_i}|} \over 2}} \right\rfloor \cdot \displaystyle{1 \over {n - \sum\nolimits_{i = 1}^m {n_i}}} \cdot \displaystyle{1 \over {{n \over h} - m}}} & \text{case 3 (type 2 or type 3)} \cr \hskip-2.5pc{\displaystyle{{|{q_i}| - 1} \over {n - \sum\nolimits_{i = 1}^m {n_i}}} \cdot \displaystyle{1 \over {{n \over h} - m}}} & \hskip-3.5pc\text{case 3 (type 4)} \cr\hskip-2pc {\displaystyle{1 \over {n - \sum\nolimits_{i = 1}^m {n_i}}} \cdot \displaystyle{1 \over {{n \over h} - m}}} & \hskip-5pc {\text{other cases}} \cr } } \right.$$

**Figure 8:**
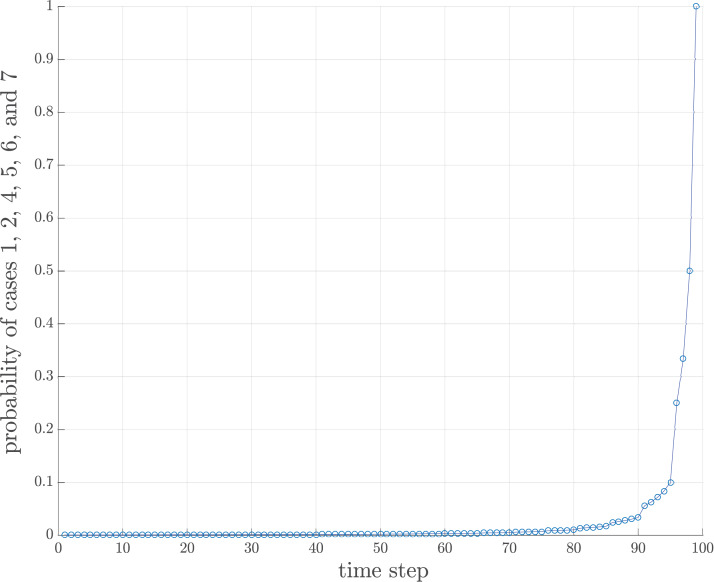
Probability of cases 1, 2, 4, 5, 6 and 7 of 100 data where *h* = 4.

Note that the value $n - \sum\nolimits_{i = 1}^m {n_i}$ and $\displaystyle{n \over h} - m$ will finally approach 1. This implies that the number of compact groups decreases and eventually there should be only one compact group. However, the time during which the probability approaches 1 depends upon the value of *h*, as shown in Fig. 8. If *h* is large, then the chance that an input chunk contains a tentative sorted sequence is also high.

**Theorem 2** *The only possible existing case to be tested for the last incoming data chunk is case 1, case 2, case 4, case 5, case 6, or case 7*.

**Proof:** The only probability approaching 1 is the probability of case 1, case 2, case 4, case 5, case 6, and case 7 as defined in Eqs. (9).*▪*

## Conclusion

This study proposed a concrete concept and practical algorithm to sort streaming numbers in the case where the total numbers overflow the actual storage. No secondary storage is involved in this constraint. The size of the working storage, *h*, for sorting is fixed throughout the sorting event. The incoming numbers are captured by new proposed data architectures in the forms of sets of single numbers and compact groups of sorted numbers. The actual order of each number with respect to the total numbers, *n*, in the streaming sequence can be correctly retrieved within *O*(*h*). The time complexity of the proposed algorithm is *O*(*n*), and the space complexity is *O*(*M*). From the experiments, it was found that the proposed algorithm can correctly and stably handle the streaming data size of at least 2.857 times larger than the size of the working storage. Furthermore, the sorted order obtained from the proposed algorithm is absolutely correct, no approximate order. In addition, each number can be directly retrieved from any *compact group* by its type. The analysis of dynamic change of used and unused working storage areas during the sorting process was also provided.

Although the proposed algorithm is primarily designed for a single processor, the proposed algorithm can be practically extended to be implemented on a multiprocessor architecture with a slight modification. In the case of a multiprocessor architecture, more than one chunk of data can simultaneously flow into the machine by one chunk per processor. The proposed algorithm can be deployed by each processor to sort each incoming chunk and to merge the final sorted results from all processors later. In fact, there are several real applications requiring this kind of sorting process where the data always overflow the working memory. Some applications are the followings:

1. Managing tremendous information inside large organizations by sorting transactions according to account numbers, locations of customers, date stamp, price or popularity of stock, ZIP code or address of mail, and so on (Sedgewick & Wayne, 2011). The proposed algorithm can reduce memory storage for keeping those data.
2. Reducing the search time of huge streaming data by sorting the data first and representing them in compact groups as implemented in *streaming data sort* algorithm.
3. Computing order statistics, quartile, decile, and percentile of big streaming data continuously flowing into an internet-scale network monitoring system and database query optimization (Buragohain & Suri, 2009).
4. Checking duplicated data for fraud detection or fake social engagement activities such as bidding on an item, filling out a form, clicking an advertisement, or making a purchase (Metwally, Agrawal & El Abbadi, 2005; Li et al., 2016).

Even though the proposed *streaming data sort* successfully sorts the streaming data under the defined constraints but some of the following further studies of streaming data sorting based on other constraints can be pursued.

1. Developing a new structure of compact group whose type can be adapted to any arbitrary different value of two temporal consecutive numbers.
2. Extending the sorting concept to cope with various data types such as a character string or a floating point number which exist in other engineering, scientific, and business problems.

## Supplemental Information

10.7717/peerj-cs.355/supp-1 Supplemental Information 1 Census-income in UCI.

10.7717/peerj-cs.355/supp-2 Supplemental Information 2 Diabetes 130-US hospitals for years 1999-2008 in UCI.

10.7717/peerj-cs.355/supp-3 Supplemental Information 3 Incident management process enriched event log in UCI.

10.7717/peerj-cs.355/supp-4 Supplemental Information 4 PM2.5 of five Chinese cities in UCI. Dew point data (Celsius Degree) in Shanghai extracted from PM2.5 data of five Chinese cities in UCI Machine Learning Repository.

10.7717/peerj-cs.355/supp-5 Supplemental Information 5 PM2.5 of five Chinese cities in UCI. Dew point data (Celsius Degree) in Guangzhou extracted from PM2.5 data of five Chinese cities in UCI Machine Learning Repository.

10.7717/peerj-cs.355/supp-6 Supplemental Information 6 PM2.5 of five Chinese cities in UCI. Dew point data (Celsius Degree) in Chengdu extracted from PM2.5 data of five Chinese cities in UCI Machine Learning Repository.

10.7717/peerj-cs.355/supp-7 Supplemental Information 7 Beijing multi-site air quality in UCI.

10.7717/peerj-cs.355/supp-8 Supplemental Information 8 PM2.5 of five Chinese cities in UCI. Dew point data (Celsius Degree) in Shenyang extracted from PM2.5 data of five Chinese cities in UCI Machine Learning Repository.

10.7717/peerj-cs.355/supp-9 Supplemental Information 9 Beijing multi-site air quality. Pressure data (hPa) in Beijing extracted from Beijing PM2.5 Data Data Set in UCI Machine Learning Repository.

10.7717/peerj-cs.355/supp-10 Supplemental Information 10 PM2.5 of five Chinese cities in UCI. Pressure data (hPa) in Guangzhou extracted from PM2.5 data of five Chinese cities in UCI Machine Learning Repository.

10.7717/peerj-cs.355/supp-11 Supplemental Information 11 PM2.5 of five Chinese cities in UCI. Pressure data (hPa) in Chengdu extracted from PM2.5 data of five Chinese cities in UCI Machine Learning Repository.

10.7717/peerj-cs.355/supp-12 Supplemental Information 12 PM2.5 of five Chinese cities in UCI. Pressure data (hPa) in Shenyang extracted from PM2.5 data of five Chinese cities in UCI Machine Learning Repository.

10.7717/peerj-cs.355/supp-13 Supplemental Information 13 PM2.5 of five Chinese cities in UCI. Pressure data (hPa) in Shanghai extracted from PM2.5 data of five Chinese cities in UCI Machine Learning Repository.

10.7717/peerj-cs.355/supp-14 Supplemental Information 14 PM2.5 of five Chinese cities in UCI. Temperature data (Celsius Degree) in Shenyang extracted from PM2.5 data of five Chinese cities in UCI Machine Learning Repository.

10.7717/peerj-cs.355/supp-15 Supplemental Information 15 PM2.5 of five Chinese cities in UCI. Temperature data (Celsius Degree) in Shanghai extracted from PM2.5 data of five Chinese cities in UCI Machine Learning Repository.

10.7717/peerj-cs.355/supp-16 Supplemental Information 16 PM2.5 of five Chinese cities in UCI. Temperature data (Celsius Degree) in Chengdu extracted from PM2.5 data of five Chinese cities in UCI Machine Learning Repository.

10.7717/peerj-cs.355/supp-17 Supplemental Information 17 Beijing multi-site air quality. Temperature data (Celsius Degree) in Beijing extracted from Beijing PM2.5 Data Data Set in UCI Machine Learning Repository.

10.7717/peerj-cs.355/supp-18 Supplemental Information 18 PM2.5 of five Chinese cities in UCI. Temperature data (Celsius Degree) in Guangzhou extracted from PM2.5 data of five Chinese cities in UCI Machine Learning Repository.

10.7717/peerj-cs.355/supp-19 Supplemental Information 19 KEGG metabolic relation network in UCI. Diameter (integer) of KEGG Metabolic Reaction Network (Undirected) Data Set in UCI Machine Learning Repository.

10.7717/peerj-cs.355/supp-20 Supplemental Information 20 KEGG metabolic relation network in UCI. Components (integer) of KEGG Metabolic Reaction Network (Undirected) Data Set in UCI Machine Learning Repository.

## Additional Information and Declarations

### Data Availability

The following information was supplied regarding data availability: Data, including permutations of random integers, are available at the UCI repository (https://archive.ics.uci.edu/ml/datasets.php). Specifically: - Beijing PM2.5 Data Data Set: https://archive.ics.uci.edu/ml/datasets/Beijing+PM2.5+Data - Census Income Data Set: http://archive.ics.uci.edu/ml/datasets/Census+Income - Covertype Data Set: https://archive.ics.uci.edu/ml/datasets/covertype - Diabetes Data Set: https://archive.ics.uci.edu/ml//datasets/Diabetes - PM2.5 Data of Five Chinese Cities Data Set: https://archive.ics.uci.edu/ml/datasets/PM2.5+Data+of+Five+Chinese+Cities - Incident management process enriched event log Data Set: https://archive.ics.uci.edu/ml/datasets/Incident+management+process+enriched+event+log - KEGG Metabolic Relation Network (Directed) Data Set: https://archive.ics.uci.edu/ml/datasets/KEGG+Metabolic+Relation+Network+(Directed) - KEGG Metabolic Reaction Network (Undirected) Data Set: https://archive.ics.uci.edu/ml/datasets/KEGG+Metabolic+Reaction+Network+(Undirected) - Buzz in social media Data Set: https://archive.ics.uci.edu/ml/datasets/Buzz+in+social+media+.
